# Gastropod Allergy: A Comprehensive Narrative Review

**DOI:** 10.3390/cimb46060355

**Published:** 2024-06-13

**Authors:** Elena Mederos-Luis, Paloma Poza-Guedes, Fernando Pineda, Inmaculada Sánchez-Machín, Ruperto González-Pérez

**Affiliations:** 1 Allergy Department, Hospital Universitario de Canarias, 38320 Tenerife, Spain; elenamederosluis@gmail.com (E.M.-L.); pozagdes@hotmail.com (P.P.-G.); zerupean67@gmail.com (I.S.-M.); 2 Food Allergy Unit, Hospital Universitario de Canarias, 38320 Tenerife, Spain; 3 Severe Asthma Unit, Hospital Universitario de Canarias, 38320 Tenerife, Spain; 4 Instituto de Investigación Sanitaria de Canarias (IISC), 38200 Tenerife, Spain; 5 Inmunotek SL Laboratories, 28805 Madrid, Spain; fpineda@inmunotek.com; 6 Allergen Immunotherapy Unit, Hospital Universitario de Canarias, 38320 Tenerife, Spain

**Keywords:** gastropods, seafood allergy, anaphylaxis, trophoallergens, dietary habits, marine ecology

## Abstract

Food allergies have increased significantly in recent decades, with shellfish being a leading cause of food allergy and anaphylaxis worldwide, affecting both children and adults. The prevalence of shellfish allergies is estimated to be approximately 0.5–2.5% of the general population, varying significantly by geographical location, age, and consumption habits. Although mollusk consumption has risen, the prevalence of mollusk allergies remains unknown. While extensive research has focused on crustacean allergies, mollusk allergies, particularly those related to gastropods, have received comparatively less attention. Clinical manifestations of shellfish allergy range from localized symptoms to life-threatening systemic reactions, such as anaphylaxis. Notably, severe bronchospasm is a predominant clinical feature in cases involving gastropods. Several allergens have been identified in mollusks, including paramyosin, tropomyosin, and sarcoplasmic calcium-binding protein. In gastropods, documented allergens include tropomyosin, paramyosin, the heavy chain of myosin, and Der p 4 amylase. Diagnosis typically involves a thorough clinical history, skin testing, in vitro quantification of immunoglobulin (Ig) E, and confirmation through an oral challenge, although the latter is reserved for selected cases. This narrative review highlights the limited research on gastropod allergy. It provides a comprehensive list of purified and recombinant allergens and discusses the applications of component-resolved diagnosis as well as current therapeutic developments.

## 1. Introduction

Seafood, including fish and shellfish, is a rich source of nutrients and antioxidants, making it a key component of the Mediterranean diet [1,2]. Known for providing essential proteins and omega-3 fatty acids, seafood offers numerous cardiovascular health benefits [3,4,5]. As a result, its consumption has surged in recent years, paralleling an increase in allergic reactions to seafood [1,2]. While shellfish includes both crustaceans and mollusks, crustacean allergies stand out as the most prevalent and extensively studied. Consequently, much of the research in this field has been focused on crustaceans, leaving studies on mollusk allergies, particularly gastropod allergies, notably sparse.

Allergens are proteins or glycoproteins capable of inducing IgE-mediated allergic reactions [6]. Typically, they are protein molecules, although carbohydrates have also been associated with some allergic capacity [7]. The molecular characteristics that determine if a molecule can be an allergen are unknown, but factors such as size, solubility, and structural stability influence their allergenic potency [6]. The part of the allergen recognized by IgE is called the epitope or antigenic determinant and consists of a series of amino acids, which can vary in size, that give rise to epitope recognition, which can be continuous or discontinuous [8].

Allergens are classified into two groups, major or minor, based on the frequency with which specific IgE is detected against them in patients sensitized to that allergenic source. An allergen is considered major if recognized by more than 50% of allergic patients and minor if recognized by less than 50%, but the frequency of allergen recognition among sensitized individuals varies across geographic regions and could also change over time [9].

Tropomyosin (TM) was the first allergen identified in *Penaeus indicus* (shrimp), and it has long been recognized as the primary allergen associated with a shellfish allergy. Interestingly, this panallergen has also been found in various invertebrate species such as cockroaches, *Anisakis simplex*, and dust mites, suggesting potential cross-reactivity between shellfish and other invertebrates [10].

However, subsequent investigations have revealed the complexity and diversity of the allergenic composition of shellfish. Several proteins shared between mollusks and crustaceans have been identified, potentially contributing to cross-reactivity. These include arginine kinase (AK), myosin light chain, sarcoplasmic calcium-binding protein (SCBP), troponin C, hemocyanin, triose phosphate isomerase, and others [10].

Current diagnostic methods may not accurately predict cross-reactivity between crustaceans and mollusks, making skin tests with fresh material—i.e., skin prick-to-prick test (PTP)—essential for detecting seafood hypersensitivity. Therefore, dietary exclusion of mollusks for shrimp-allergic patients should rely on a personalized clinical history, diagnostic in vivo and in vitro tools, and ultimately oral challenge tests.

The aim of this paper is to collate the limited scientific literature on gastropod allergy and examine the currently available clinical and immunological data.

## 2. Epidemiology of Shellfish Allergy

Food allergy (FA) refers to an adverse immune system reaction to certain foods [11]. In recent years, the prevalence of FA has significantly increased, affecting an estimated 3.5–4% of the global population [12,13,14]. The rising consumption of shellfish in recent years has heightened the risk of allergic and toxic reactions, presenting with a variety of symptoms that can be challenging to define. Shellfish are a leading cause of FA and anaphylaxis worldwide, with prevalence estimated at approximately 0.5–2.5% of the general population. This prevalence varies based on geographical location, age, and consumption habits [12]. Recent research by Gelis and colleagues suggested that the prevalence of shellfish allergies range from less than 1% to 10.3%, depending on geographical area [10]. In Europe, there is also a significant variation in the prevalence of shrimp allergy across different studies, ranging from 10.2% in Italy, 2.8% in Iceland, to 0.3% in Denmark [15,16,17]. For instance, in Spain, shellfish is the third most common cause of FA in adults over 15 years old, with cases increasingly reported at younger ages, following milk, egg, fruit, and fish allergies [18]. Moreover, the shellfish allergy is one of the leading causes of FA in many Asian countries, such as Thailand, Taiwan, Hong Kong, Vietnam, and Singapore, where shellfish is frequently consumed [19]. The coastal regions of Asia are prominent consumers of mollusks, while Southern Europe, particularly Spain, favors cephalopods and other shellfish. Japanese diets feature higher quantities of squid, whereas Italians, French, Portuguese, and Spaniards consume significant amounts of terrestrial snails [12]. Consequently, awareness of mollusk allergies is growing, although its prevalence remains uncertain [13]. Additionally, the allergy to mollusks, particularly gastropods, has received limited study.

## 3. Classification of Shellfish

**Figure 1:**
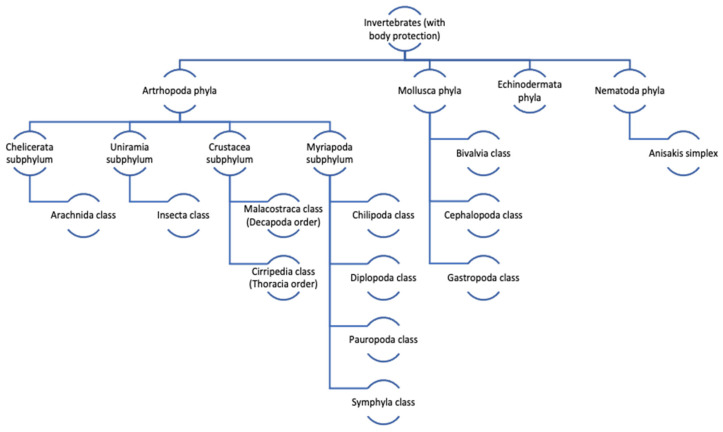
Taxonomic tree of edible shellfish species within *Crustacea subphylum* and the *Mollusca phylum* (adapted from [20]).

The term shellfish is used for both crustaceans and mollusks. Mollusks represent the largest marine phylum, with around 85,000 described species [12]. Shellfish belong to the Invertebrate Kingdom Eumatozoa, which is divided into three *phyla* as follows: Mollusca, Athropoda, and Echinodermata. Athropoda contains the class Crustacea. The Mollusca phylum is divided into eight classes, but only three are significant for human consumption, namely cephalopods (cuttlefish, squid, octopus), bivalves (clams, cockles, mussels, blue mussels, scallop, oyster), and gastropods (limpets, conchs, periwinkles, sea slugs, whelks, snails, and abalone) [14] (Figure 1).

## 4. Clinical Symptoms of Shellfish Allergy

Food allergies (FA) may be classified into the following three categories based on the involvement of immunoglobulin (Ig) E in the immune response: IgE-mediated, non-IgE-mediated, and mixed IgE- and non-IgE-mediated reactions. These immune responses may elicit type I hypersensitivity (IgE-mediated), type III, type IV hypersensitivity (non-IgE-mediated), or a combination of IgE and cellular mechanism (mixed) [11]. Normally, IgE-mediated responses occur rapidly, within two hours of ingestion, presenting with clinical symptoms such as urticaria, angioedema, abdominal pain, nausea, vomiting, or respiratory issues as bronchospasm, laryngeal edema, and/or anaphylaxis [11]. Additionally, patients may exhibit localized, self-limited symptoms in the oropharyngeal mucosa due to shellfish cross-reactivity with inhalant allergens such as house dust mites (HDM) and tropomyosin (TPM), described as the mite–shellfish oral allergy syndrome [21].

**Figure 2:**
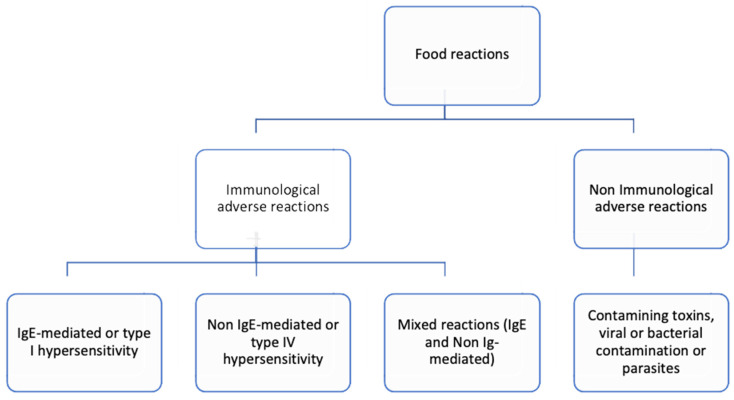
Classification of food reactions based on the mechanism involved.

Non-IgE-mediated reactions, progressively recognized in children, usually manifest a few hours or days after subjection to the offending allergen. These reactions include food protein-induced enterocolitis syndrome (FPIES), food protein-induced enteropathy (FPE), and food protein-induced allergic proctocolitis (FPIAP) [1,22,23,24]. In contrast to soybean and/or dairy products FPIES, a later onset of longer and more persistent symptoms and the likelihood of tolerating fish species alternative to the offending fish are characteristic attributes of acute fish and shellfish FPIES [1]. Also, contaminating toxins, viral and bacterial contamination, or parasites can cause adverse symptoms such as vomiting, fever and diarrhea after the ingestion of shellfish. These clinical manifestations typically emerge several hours after ingestion [25] (Figure 2).

Symptoms of FA are triggered by food proteins that activate the immune system, leading to an increase in IgE levels. Typical symptoms include itching and swelling of the mouth and throat (allergic oral syndrome), as well as potentially life-threatening anaphylaxis [12]. The clinical manifestations of shellfish allergy can vary widely and differ among individuals. It typically results in moderate to severe reactions, characterized by sensitization that often persists throughout life, with avoidance being the only effective treatment [12]. Exposure to shellfish allergens can occur through ingestion, inhalation, or skin contact. Symptoms may include itching, hives (urticaria), swelling (angioedema), respiratory symptoms (shortness of breath, coughing, wheezing, rhinitis), gastrointestinal symptoms (nausea, vomiting, diarrhea, abdominal pain), and cardiovascular symptoms (hypotension) [12]. In severe cases, life-threatening reactions can occur. Allergic reactions to shellfish can be unpredictable. While type I reactions typically occur within the first hour, there have been reported cases where the symptoms appeared up to 8 h after the ingestion of limpet and abalone [26].

## 5. Clinical Symptoms of Gastropod Allergy

The gastropod allergy is notable for its severe symptoms, particularly marked by pronounced bronchospasm, which serves as a hallmark of reactions within this category. While severe asthma can manifest in reactions to other shellfish, it is particularly distinctive in cases involving gastropods [26,27,28,29,30,31,32].

### 5.1. Terrestrial Snail

Snail hypersensitivity was initially reported by Palma Carlos et al. in 1985 [33]. Subsequent studies have further elucidated this phenomenon. Four years later, De la Cuesta and colleagues presented findings from 10 patients, 80% of whom reported respiratory symptoms, with 2 experiencing symptoms after consuming limpet and snail. Interestingly, despite all the described patients tolerating the ingestion of both cephalopods and bivalves, which belong to different phylogenetic lines, there was a lack of data regarding the concomitant tolerance to crustaceans and the prevalence of comorbid asthma [27]. In 1996, Van Ree et al. reported 28 subjects who experienced asthmatic episodes after consuming snails, with 2 cases resulting in anaphylactic reactions. Some of these patients also reported similar respiratory symptoms after ingesting limpets. Furthermore, 23 out of those 28 subjects presented symptoms within 5–60 min after snail ingestion, while the remaining subjects experienced symptoms 1–5 h post ingestion. There was also a lack of data regarding the tolerance to crustaceans or other mollusks. Notably, all subjects presented with dust mite allergic rhinitis and asthma, suggesting a potential co-sensitization [28]. Guilloux, Vuitton, and coworkers reported in 1998 that seven patients experienced respiratory symptoms and anaphylaxis after consuming terrestrial snails. The diagnosis was established through skin tests and specific IgE against snails. Similar to the previous findings, there was limited data concerning the tolerance of other mollusks or crustaceans. All the patients were allergic to *Dermatophagoides pteronyssinus* (*D. pteronyssinus*), with five of them being asthmatic [34]. In 2005, Lourenço Martins et al. also identified 60 allergic patients with specific IgE to *Helix aspersa* (*H. aspersa*). Among them, six developed asthma after consuming snails, with symptoms appearing 15 min to 3 h post ingestion. Once again, data regarding the tolerance of other mollusks or crustaceans were scarce. Of the 60 patients, 18 suffered from asthma, 36 from rhinitis and asthma, and 3 from rhinitis, 2 from atopic dermatitis, and 1 from irritative cough. Notably, 56 patients were allergic to *D. pteronyssinus*, suggesting a potential cross-reactivity between species [29].

### 5.2. Abalone

In 1990, Morikawa documented a case of anaphylaxis associated with abalones, highlighting specific IgE-mediated hypersensitivity to these shellfish confirmed through clinical history, prick skin tests, and radioallergosorbent (RAST) tests. Further analysis using RAST inhibition techniques revealed cross-antigenicity between GKL, abalone, and keyhole limpet hemocyanin. However, data regarding the tolerance of other mollusks or crustaceans and the prevalence of asthmatics remained scarce [35]. Lopata et al. (1997) reported on 38 patients, with 66% experiencing symptoms within 2 h and 34% between 2 and 7 h after ingesting abalones. Respiratory and cutaneous reactions were predominant in this cohort. The diagnosis was confirmed through skin tests and positive RAST responses, with 58% of patients having atopic diseases. Yet there is a lack of information concerning the tolerance of other shellfish and the prevalence of asthma within this group [26].

### 5.3. Limpet

In 1991, Carrillo and colleagues reported two cases of allergic reactions following limpet ingestion. One of the patients exhibited diffuse urticaria, angioedema, status asthmaticus, and severe hypotension 60 min post ingestion, while the other experienced abdominal cramps, dysphagia, diffuse erythema, dysphonia, severe bronchospasm, loss of consciousness, and respiratory arrest 40–60 min following the ingestion of limpets. Both cases tested positive for cooked limpet extract on the skin prick-to-prick tests and presented positive IgE against limpet. Remarkably, they tolerated other mollusks and crustaceans and presented rhinitis and asthma due to dust mite exposure [30]. Later in 1994, Carrillo and colleagues reported on six subjects who developed severe bronchospasm 30 to 120 min after consuming limpets. The diagnosis was confirmed by a skin prick-to-prick test with limpet and specific IgE. While all the patients were sensitized to *D. pteronyssinus*, the number of asthmatics was unspecified. Data regarding the tolerance of other mollusks or crustaceans were not available [36]. Azofra and Lombardero (2003) presented five cases of anaphylaxis following limpet ingestion, with symptoms occurring between 10 and 90 min post ingestion, with bronchospasm being a prominent manifestation. The diagnosis was carried out by a skin test positive to limpet with positive IgE specific to limpet. They tolerated crustaceans and other mollusks, and all had house-dust-mite-related asthma [31]. In 2008, Gutiérrez-Fernández et al. reported one patient with urticaria and angioedema 30–45 min after the ingestion of limpet, confirmed by a skin prick-to-prick test with raw and cooked limpet and specific IgE determinations for raw and cooked limpet. This patient tolerated crustaceans and other mollusks (cephalopods and bivalves) and only presented symptoms of rhinitis due to dust mite sensitization [37]. Azofra (2017) recruited 11 patients with a gastropod allergy, where the diagnosis was made based on a clear history of adverse reaction suggestive of IgE-mediated allergy after eating gastropods, along with positive skin test results with the same gastropod. Their symptoms included systemic reactions such as urticaria, angioedema, bronchospasm, abdominal symptoms, and hypotension. While some patients showed positive results in the skin tests specific to crustaceans, all of them tolerated the crustaceans. These patients were predominantly dust-mite-allergic asthmatics who frequently developed serious bronchospasm or anaphylaxis immediately after eating gastropods as reported [13].

In 2023, our group reported 16 patients with a confirmed limpet allergy exhibiting good tolerance to other shellfish. Contrary to the descriptions of other shellfish allergies, clinical symptoms typically appeared later (up to an average of 121 min) and were often severe, including anaphylaxis (62.5%) or asthma alone (31.25%). All the patients also had a medical history of rhinoconjunctivitis, and 50% (8/16) had asthma due to dust mite allergy [32]. Upon analysis of the clinical presentations of allergic reactions following gastropod ingestion, the delayed onset of symptoms compared to other shellfish classes was remarkable.

## 6. Diagnostic Tools

### 6.1. In Vivo Diagnosis

Currently, the diagnostic tools for gastropod allergies are limited. The primary focus lies on conducting a thorough medical history, which includes understanding the patient’s clinical background, the symptoms experienced, and the type of reaction observed, alongside a comprehensive physical examination.

#### 6.1.1. Skin Testing

Testing can be performed using either commercial whole allergen extracts or fresh allergens, such as PTP tests. However, several factors must be taken into consideration when explicating these results, including the potential for cross-reactivity among shellfish, house dust mites, and cockroaches; variations in test protocols; lack of standardization in diagnostic allergen extracts; and the effects of shelf-life and reagent stability on the sensitivity and specificity of ordinary skin tests [38]. For instance, in a study including both children and adults, the analysis of five commercial shellfish SPT extracts revealed significant variability in IgE reactivity during immunoblotting, ranging from 59% to 79% sensitivity. Following sodium dodecyl sulfate polyacrylamide gel electrophoresis (SDS-PAGE), these researchers also observed a noticeable absence of protein bands in commercial crude preparations compared to freshly prepared in-house shrimp extracts [38].

In the case of gastropods, given the lack of commercial extracts for limpet and abalone, additional prick-to-prick skin tests are often performed using the natural food items on the volar side of each subject’s arm, with both raw and cooked presentations of the implicated gastropod. A skin test is considered positive if a wheal with a diameter equal to or greater than 3 mm appears, in comparison to a negative control (saline solution, 0 mm), with a positive response to histamine (10 mg/mL) [39]. Wheal diameters are measured 20 min immediately after testing.

#### 6.1.2. Oral Food Challenge

Oral food challenges (OFCs) come in three types, namely open, single-blind, and double-blind, with the latter considered as the gold standard for diagnosing shellfish allergy. However, OFCs are resource-intensive and carry a risk of severe, potentially life-threatening allergic reactions [40]. In clinical practice, if the medical history strongly suggests shellfish allergy based on reaction severity, and if skin tests and/or specific IgE tests are positive, an avoidance diet may be recommended [1,11,41]. Conversely, if skin and serological tests yield negative results, an open OFC is typically advised to confirm the diagnosis, with an emphasis on individualizing each case [1]. 

### 6.2. In Vitro Diagnosis

Since the discovery of IgE, technology has provided new laboratory tools to quantify IgE antibody levels in the serum of the allergic patient. Quantitative immunoassays for IgE antibodies serve as both a complement to skin tests and an essential component of diagnostic precision [42]. The measurement of allergen-specific IgE antibodies occurs amidst the presence of other antibodies of the same isotype, alongside allergen-specific IgE antibodies and various isotypes specific to the same allergen. This necessitates specific recognition by allergen binding sites (Fab) and epitopes within the same test. Hence, allergenic extracts utilized in these assays must undergo a thorough characterization to ensure the generation of accurate and reproducible data in clinical allergy research. The allergosorbent (solid-phase) reagent stands as the pivotal component of the assay, imparting specificity to the IgE antibody assessment. To enhance the antibody-binding capability beyond traditional paper discs, a range of carbohydrate-based allergosorbents like microcrystalline cellulose and agarose, have historically been employed in research settings. However, the most notable advancement in clinical trials came with the progress of an encapsulated hydrophilic polymer to which the allergen adhered. Configured as a small cup, this polymer, termed CAP, revolutionized the field. In this way, immunoCAP was used to determinate the presence of IgE against common aeroallergens, shellfish allergens, and against terrestrial snail (the only gastropod-specific IgE available at this moment) in a singleplex configuration.

IgE antibody tests can be performed as singleplex or monoplex (single) assays; with reference to laboratory methods in which one analyte is measured per analysis, multiallergen (<10) and multiplex (>100 allergen specificity) assays permit more than one analyte to be detected and quantified in a single assay analysis, with all the same design and performance characteristics, such as with the ISAC, ALEX2 and Euroline platforms [43,44,45]. In brief, ALEX^®^ (MacroArray Diagnostics, Vienna, Austria) is an advanced multiplex array consisting of 295 reagents, comprising 178 molecules and 117 extracts of airborne allergens and cross-reactive food allergens. It boasts the unique capability of simultaneously measuring the concentration of serum sIgE (within a test range of 0.3–50 kUA/L) and total IgE (within a test range of 1–2500 kU/L). This innovative system pairs different allergens and components onto polystyrene nano-beads, which are subsequently deposited onto a nitrocellulose membrane, following a methodology previously documented in published literature [46]. A total of five shellfish molecular allergens were investigated as follows: Pen m 1, Pen m 2, pen m 3, Pen m 4 and Cra c 6.

## 7. Overview of Mollusk Allergens

This section provides a brief overview of the biochemical properties and protein structure of the most relevant identified mollusk allergens.

### 7.1. Lepetellida

#### 7.1.1. *Haliotis laevigata* and *Haliotis rubra*

*Hal l 1*: A single reference is provided below concerning information related to its protein structure, along with some annotations regarding cases related to this sensitization. Specifically, five out of nine individuals with case histories of allergy to consumption of crustacean shellfish, and one after handling prawns, were regarded as positive. All patients showed a positive (≥0.35 kU) ImmunoCAP^®^ result for oyster. Additionally, tests confirmed the binding of IgE to abalone tropomyosin in reducing immunoblots [47].

#### 7.1.2. *Haliotis midae*

*Hal m 1*: Abalone allergens consist of heat-stable proteins with molecular weights of 38 and 49 kDa, subsequently identified as HalIn-1 in accordance with the International Union of Immunological Societies allergen nomenclature regulation. Former research suggests a discernible clinical and immunologic diversity among patients exhibiting reactivity to abalone [26]. Please note that allergic reactions to abalone have been previously addressed in Section 5.2. of the present manuscript.

### 7.2. Neogastropoda

#### *Rapana* *venosa*

*Rap v 2*: An allergenic protein weighing 99 kDa, extracted from *Rapana venosa* (*R. venosa*), was identified as paramyosin (PM) through mass spectrometry. Despite its importance as a structural protein in molluscan muscles, limited information exists on PM’s allergenic properties in mollusks. These findings revealed that *R. venosa* PM can bind specific IgE antibodies from sea-snail-allergic patients, with its binding activity being reducible by thermal treatment. The full-length cDNA sequence of *R. venosa* PM, consisting of 859 amino acids, exhibits significant homology across molluscan species. Our analyses using circular dichroism, Fourier transform infrared, and 2D/3D structure assessments demonstrate that both PM and tropomyosin are conserved proteins, predominantly comprising α-helix structures. These insights contribute to a deeper understanding of anaphylactic reactions in sea-snail-allergic individuals and advancements in allergy diagnosis [48].

### 7.3. Ostreida

#### 7.3.1. *Crassostrea* *angulate*

*Cra a 1*: Tropomyosin (TM), a key allergen in *Crassostrea angulata*, was purified and identified via mass spectrometry. TM was then cloned and expressed, revealing a sequence of 852 bp encoding 284 amino acid residues. Circular dichroism, digestion assays, inhibition enzyme-linked immunosorbent assays, and basophil activation tests indicated that recombinant TM exhibited similar physicochemical and immunological properties to native TM. Additionally, two conformational mimotopes and ten IgE linear epitopes were identified. Varying degrees of cross-reactivity were observed between *C. angulata* TM and TMs from eight other shellfish species, likely due to three conserved epitope regions. These insights may aid in the molecular diagnosis of oyster allergies and cross-reactivity among shellfish [49].

*Cra a 2*: Arginine kinase (AK) was identified as a novel allergen in *Crassostrea ngulate*. The primary AK sequence was cloned, encoding 350 amino acids, and recombinant AK (rAK) was produced. Immunodot assays, secondary structure analyses, and digestive stability tests showed that both native AK and rAK had similar IgG/IgE-binding activities and physicochemical properties. Serological analysis of 14 oyster-sensitive individuals revealed AK’s cross-reactivity among oysters, shrimp, and crabs [50].

*Cra a 4*: A 20 kDa protein was purified from oysters and identified as sarcoplasmic calcium-binding protein (SCP) through LC-MS/MS. A 537 bp open reading frame was obtained from oyster SCP total RNA, encoding 179 amino acids, and expressed in *Escherichia coli*. Circular dichroism results, digestion assays, and inhibition ELISA demonstrated that recombinant SCP (rSCP) had similar physicochemical properties and IgG-binding activity to native SCP, while showing stronger IgE-binding activity. Cross-reactivity and sequence homology varied among shellfish species. These findings offer new insights into shellfish allergens and can facilitate the in vitro diagnosis of oyster sensitization (GenBank: QIJ32297.1).

#### 7.3.2. *Crassostrea* *gigas*

*Cra g 1*: From oysters, a 20 kDa protein was purified and identified as sarcoplasmic calcium-binding protein (SCP) via LC-MS/MS. Subsequently, an open reading frame of 537 base pairs was isolated from oyster SCP total RNA, encoding 179 amino acids, and expressed in *Escherichia coli*. Comparative analyses including circular dichroism, digestion assay, and inhibition ELISA revealed that the recombinant SCP (rSCP) shared analogous physicochemical properties and IgG-binding activity with native SCP. Additionally, rSCP demonstrated heightened IgE binding activity, along with varying degrees of cross-reactivity and sequence homology observed among shellfish species. These findings offer fresh insights into shellfish allergens, potentially enhancing the in vitro diagnosis of oyster-sensitized patients [51].

#### 7.3.3. *Saccostrea* *glomerata*

*Sac g 1*: Mass spectrometry identified IgE-reactive proteins in Sydney rock oysters, leading to the cloning, sequencing, and designation of a novel major oyster tropomyosin allergen as Sac g 1 by the IUIS. Oyster extracts exhibited the highest IgE cross-reactivity with other mollusks, with the weakest cross-reactivity observed with mussels [52].

### 7.4. Stylommatophora

#### *Helix* *aspersa*

*Hel as 1*: The cloned tropomyosin from brown garden snails exhibits significant similarity to tropomyosins from other edible mollusks (with identities ranging from 84% to 69%), as well as to those found in arthropods (with identities ranging from 65% to 62%), and comparatively less resemblance to vertebrate tropomyosins (with an identity of 56%). Tropomyosin elicited an immune response in 18% of the sera obtained from patients with snail allergies. Inhibition experiments utilizing both natural and recombinant tropomyosins revealed varying levels of cross-reactivity among invertebrate tropomyosins. Sera from individuals allergic to snails recognized tropomyosins in the extracts from both mollusks and crustaceans [53].

### 7.5. Teuthida

#### *Todarodes* *pacificus*

*Tod p 1*: The isolated allergen from squid is a heat-stable protein weighing 38 kDa. Immunoblotting confirmed the binding of IgE antibodies to the purified squid allergen. Cross-reactivity was observed between major allergens of squid and shrimp, as evidenced by the sera from patients allergic to either squid or shrimp, or by allergen-specific monoclonal antibodies. Sequence analysis of the major squid allergen revealed significant homology with tropomyosin from the blood fluke planorbid (*Biomphalaria glabrata*), a common vector snail of *Schistosoma mansoni*. This 38 kDa protein, identified as Tod p 1 according to the regulations of the International Union of Immunological Societies allergen nomenclature (WHO/IUIS), is a principal allergen of the squid *Todarodes pacificus* and is believed to be squid muscle protein tropomyosin [54].

## 8. Focus on Gastropod Allergens: State of the Art

Allergy to gastropods is inadequately documented in the scientific literature, with only a limited number of reported cases. This scarcity of documentation may be attributed to the localized consumption of this type of shellfish, primarily in regions such as Spain, France, Italy, and Portugal. Additionally, the coastal regions of Asia are known for their significant consumption of mollusks, contributing to the prevalence of gastropod allergy in these areas [12].

Unlike other shellfish allergies, reactions to gastropods often manifest later and tend to be more severe, frequently involving severe respiratory symptoms. Due to the potential severity of these reactions, it is advisable for individuals experiencing suggestive symptoms to avoid not only ingesting gastropods but also inhaling cooking vapors or encountering these shellfish.

At present, our diagnostic capabilities for gastropod allergy are limited. We rely on the commercial snail extract available for conducting skin prick tests and specific IgE testing against snail allergens. Unfortunately, there are no commercial extracts or specific IgE available for limpet and/or abalone, necessitating the use of fresh raw and cooked food for skin prick tests. Additionally, we have access to the ALEX^®^ technique, which includes a panel of five shellfish allergens (Pen m 1, Pen m 2, Pen m 3, Pen m 4 y Cra c 6). However, further studies are required to ascertain the reliability of these allergens for diagnosing gastropod allergy in our patients. It is worth noting that our group has presented our preliminary findings at the EAACI 2023, indicating some degree of allergen recognition among a subset of patients using the ALEX^®^ technique [32].

While the gold standard for diagnosing food allergies remains the oral tolerance test, in many cases, a comprehensive medical history combined with positive results from a skin prick test or specific IgE testing may provide sufficient confirmation. This approach is particularly applicable given the often-severe reactions experienced by patients following the ingestion of certain types of shellfish. Additionally, in mild cases, many patients may opt out of undergoing the oral tolerance test. Among the various subgroups within a shellfish allergy, the crustacean allergy stands out as the most prevalent and extensively studied. Consequently, much of the research in this field has been focused on crustaceans.

Within the Gastropoda class, allergy to terrestrial snails has emerged as a significant focus in scientific literature, drawing extensive study. Research by Guilloux et al. [34] and Van Ree et al. [28] has underscored the notable cross-reactivity between dust mites and terrestrial snails. Notably, several allergens implicated in this cross-reactivity, including Der p 4, p 5, p 7, and hemocyanin, have been identified [34]. Interestingly, while tropomyosin does not appear to play a role in this cross-reactivity, the RAST assays conducted by these researchers have revealed compelling evidence. They found that the reactivity of snail IgE is inhibited by dust mite extract, suggesting that dust mites may serve as the primary sensitizing agent. This finding adds depth to our understanding of allergenic interactions between dust mites and terrestrial snails, shedding light on the potential mechanisms underlying snail allergy [34].

In 2005, Lourenço Martins et al. conducted a study involving 60 allergic patients with specific IgE to *H. aspersa*. Interestingly, they found that specific IgE concentrations did not correlate with the number of recognized allergens or allergic responsiveness among the patients studied. Notably, only 1 individual out of 21 recognized a 37 kDa protein from *H. aspersa* extract. The study identified the heavy chain of myosin (225 kDa) as one of the two major allergens, found in 13 and 18 out of 21 patients, respectively. Additionally, five patients who experienced clinical symptoms after snail ingestion recognized at least one major allergen from *H. aspersa* extract > 208 kDa. This suggests that the protein domains involved in the allergic response may be present in the three-dimensional structure of snail myosin [29].

In 2016, Misnan et al. conducted a study investigating the effects of thermal treatments on major and minor allergens of sea snails (*Cerithidea obtusa*). They found that fried snails exhibited the most significant reduction in both the number of bands and their intensities compared to other cooking methods. The study revealed the presence of thermolabile proteins within a wide range of molecular weights, including those ranging from 10 to 17 kDa, 25 to 30 kDa, 40 to 74 kDa, and some high molecular weight bands (124–250 kDa) in all the cooked extracts. Interestingly, most snail proteins were sensitive to heat, except for a few bands at 17, 18, 20, 33, 42, and 124 kDa, which demonstrated resistance to heat denaturation. Notably, the 33 kDa protein was identified as the most significant major allergen in *C. obtusa*, believed to be tropomyosin [55]. This study sheds light on the impact of thermal processing on the allergenicity of sea snails, providing valuable insights for food safety and allergen management practices.

In 1997, Lopata et al. documented 13 subjects who experienced symptoms up to 7 h after consuming abalones [26]. Contrary to the findings of Guilloux et al. [34], a RAST inhibition study conducted by Lopata et al. did not reveal cross-reactivity between abalone and dust mites. Interestingly, they identified a single 49 kDa protein, recognized by the serum IgE of five patients, but it was not related to abalone tropomyosin (38 kDa) [26]. This study highlights the complexity of allergenic proteins in abalones and suggests the presence of unique allergens unrelated to tropomyosin.

In the Canary Islands, limpet consumption is prevalent in the local diet. In 1991, Carrillo et al. [30] were the first to document two cases of anaphylaxis following the ingestion of limpets. Subsequently, in 1994, Carrillo et al. expanded their study to include six patients, concluding that limpets could pose a potentially serious allergen for individuals sensitized to *D. pteronyssinus* [36]. Conversely, in 2003, Azofra et al. described five patients with a history of limpet allergy and identified a 75 kDa protein in their cases that could be related to Der p 4 amylase [31]. This highlights the variability in allergenic proteins associated with limpet allergy and underscores the importance of continued research in this area for accurate diagnosis and management of allergic reactions.

In our local study, spanning a period of 12 months, we enrolled a total of 16 patients who were conclusively diagnosed with limpet allergy [32]. Among these individuals, only four patients exhibited positive results for various shellfish allergens using ALEX^®^, including Cra c 6 (Troponin C), while one patient tested positive for Pen m 1, Pen m 3, and Pen m 4. Further analysis through Western blotting unveiled that the combined sera from these patients recognized a couple of bands ranging between 36 and 40 kDa in both raw and cooked limpet extracts. Additionally, a 37 kDa band was identified in cooked shrimp extract, consistent with tropomyosin. On an individual basis, some patients also identified bands within the range of 25–40 kDa and 50–200 kDa, with these bands being more pronounced in the raw extracts. This stands in contrast to observations in shrimp extracts, where bands typically become more apparent in the cooked form [32]. This observation could be attributed to the effects of thermal treatments on major and minor allergens, as described by Misnan et al. [55] (Table 1).

## 9. Limitations

Currently, the diagnosis of a gastropod allergy faces significant challenges due to a lack of diagnostic tools. Additionally, the scientific literature concerning gastropod allergies is sparse, with only a few reported cases documented. Furthermore, an important consideration is the emergence of novel foods such as insects, which are increasingly incorporated into certain diets. These novel food sources may pose a risk of cross-reactivity due to shared proteins with other invertebrates [58].

## 10. Future Perspective and Conclusions

The available studies on limpets are scarce, which poses significant limitations in the diagnosis of limpet allergy. Currently, the lack of both a specific molecular diagnosis for this gastropod and its commercial extract restricts the diagnostic procedure, particularly in regions like ours, the Canary Islands, where limpet consumption is prevalent compared to other geographical areas. There is an urgent need to optimize the study and the diagnosis of limpet allergies to enhance the performance of allergy studies and improve the accuracy of diagnosis. By doing so, we aim to reduce the unnecessary avoidance of limpets and provide better management options for our patients.

Despite numerous proteins being described, tropomyosin and Der p 4 amylase are often mentioned in the context of gastropod allergy and potential cross-reactivity with dust mites, based on their approximate molecular weights. However, further studies are essential to precisely identify the specific proteins involved, ascertain their allergenic properties, and determine their clinical significance. Many studies have indicated cross-reactivity with dust mites, as evidenced by the presence of proteins in both extracts with similar molecular weights and positive results in inhibition RAST tests, where *D. pteronyssinus* appears to be the sensitizing agent [34,59]. However, in populations like ours, it is conceivable that proteins shared between dust mites and gastropods may exist, suggesting the possibility of co-sensitization. Nevertheless, comprehensive series and molecular studies are required to fully elucidate this complex matter and provide a better understanding of the mechanisms underlying the cross-reactivity and allergenicity between dust mites and gastropods.

Cross-reactivity among different gastropods or between gastropods and other shellfish remains understudied. Additionally, recent research suggests that O-glycosylation may play a role in patients experiencing anaphylaxis due to snails and allergy to *Artemisia vulgaris* [60]. This finding highlights the complexity of allergenic mechanisms and underscores the importance of further investigation into the role of glycosylation and its implications for shellfish allergy management and diagnosis.

In 2008, the first IgE-mediated anaphylactic reaction to the therapeutic monoclonal antibody Cetuximab was identified in a patient with meat allergy [61,62]. This IgE antibody is specific to alpha-Gal, as demonstrated by the analysis of neoglycoprotein (e.g., human serum albumin-alpha-Gal) conjugates [63]. It is known that allergens from various sources, such as invertebrates and parasites like helminths, present common carbohydrate structures. These glycans, known as classical carbohydrate determinants (CCDs), have well-established IgE-binding properties.

The classical CCDs feature a non-human IgE-binding monosaccharide unit, typically xylose, while fucose linked in CCDs is predominantly a human monosaccharide. Both fucose and xylose residues have been identified as contributors to IgE binding and cross-reactivity [7,64]. This recent insight has led to the categorization of two primary groups of subjects with IgE, namely group A, characterized by antiglycan IgE, and group B, exhibiting IgE against the peptide fraction of an allergen. Patients in group B are relatively well understood in clinical practice and can often be diagnosed using existing testing methodologies. However, group A poses a diagnostic challenge due to IgE cross-reactivity against cross-reactive carbohydrate determinants (CCDs). Within group A, the following two subgroups are delineated: group A1 consists of patients with antiglycan IgE and clinically significant allergy symptoms, such as IgE against galactose-alpha-(1,3)-galactose (alpha-Gal), while group A2 includes patients with antiglycan IgE but either no allergy symptoms or minor symptoms, primarily directed against CCDs. For antiglycan IgE diagnosis, in addition to CCD diagnosis, recent advancements include commercial systems for detecting antiglycan IgE against alpha-Gal [63]. Therefore, the consideration of carbohydrates and glycosylation could be pivotal in allergy diagnosis and management.

Hence, it is imperative to acquire commercial extracts with enhanced sensitivity to effectively detect patients allergic to gastropods. Additionally, efforts should focus on identifying allergenic proteins from various consumable gastropods to incorporate them into diagnostic tests. This approach aims to ascertain whether the coexistence of dust mite and gastropod shellfish allergies, as well as allergy to other shellfish groups, arises from common proteins (cross-reactivity) or mere co-sensitization, thus providing insight into the actual probability of cross-reactivity between these groups. Allergic reactions to gastropods tend to be severe, posing potential life-threatening risks to affected individuals. Consequently, it is crucial to offer comprehensive health education, prescribe, and provide guidance on the use of epinephrine auto-injectors and other necessary medications. However, addressing these challenges consumes significant time and resources. Advancements in the characterization of gastropod allergens are paramount. These advancements not only facilitate the development of accurate diagnostic methods but also contribute to a deeper understanding of this condition, thereby improving overall knowledge and management of gastropod allergies.

## Figures and Tables

**Table 1:** Literature Review: Studies on Gastropods. Unk: Unknown.

Author	Year of Publication	Gastropod	Allergen	Molecular Weight (kDa)	References
Morikawa et al.	1990	Abalone	Unk	Unk	[35]
Carrillo et al	1991; 1994	limpet	Unk	Unk	[30,36]
Van Ree et al.	1996	Snail	Unk	Unk	[28]
Lopata et al.	1997	Abalone	Unk	49	[26]
Guilloux et al. Muddaluru et al.	1998 2021	Snail	Der p 4	18	[34,56]
			Der p 5	13	
			Der p 7	14	
			Hemocyanin	75–85	
Azofra et al.	2003	limpet	Der p 4 amylase	75	[31]
Laurenço Martins et al.	2005	Snail	Tropomyosin	37	[29]
			Heavy chain of myosin	225	
			3-dimensional structure of snail myosin	>208	
Suzuki et al.	2011	Abalone	Paramyosin	89	[57]
Misnan et al	2016	Snail	Tropomyosin	33	[55]
Azofra et al.	2017	Limpet	Actin	46–47	[13]
Mederos-Luis et al.	2023	limpet	Unk	36–40	[32]
			Unk	50–200	

## Data Availability

The data that support the findings of this study are available from Servicio Canario de la Salud; however, restrictions apply to the availability of these data, which were used under license for the current study, and so are not publicly available. Data are, however, available from the authors upon reasonable request and with the permission of Servicio Canario de la Salud.

## References

[1] Giovannini M., Beken B., Buyuktiryaki B., Barni S., Liccioli G., Sarti L., Lodi L., Pontone M., Bartha I., Mori F. (2023). IgE-Mediated Shellfish Allergy in Children. Nutrients.

[2] Xu L., Cai J., Gao T., Ma A. (2022). Shellfish consumption and health: A comprehensive review of human studies and recommendations for enhanced public policy. Crit Rev Food Sci Nutr..

[3] Venter C., Smith P.K., Arshad H. (2022). Dietary strategies for the prevention of asthma in children. Curr. Opin. Allergy Clin. Immunol..

[4] Venter C., Meyer R.W., Greenhawt M., Pali-Schöll I., Nwaru B., Roduit C., Untersmayr E., Adel-Patient K., Agache I., Agostoni C. (2022). Role of dietary fiber in promoting immune health-An EAACI position paper. Allergy.

[5] Martínez-González M.A., Gea A., Ruiz-Canela M. (2019). The Mediterranean Diet and Cardiovascular Health. Circ. Res..

[6] Beitia Mazuecos J.M., Cano G., del Mar M., Moreno Fernández A., Pelta Fernández R. (2023). Guía Práctica de Alergología.

[7] Van Ree R., Cabanes-Macheteau M., Akkerdaas J., Milazzo J.P., Loutelier-Bourhis C., Rayon C., Villalba M., Koppelman S., Aalberse R., Rodriguez R. (2000). β (1,2)-xylose and α (1,3)-fucose residues have a strong contribution in IgE binding to plant glycoallergens. J. Biol. Chem..

[8] Taylor S.L., Lemanske R.F., Bush R.K., Busse W.W. (1987). Food allergens: Structure and immunologic properties. Ann. Allergy.

[9] García-Figueroa B.E., Díaz-Perales A., Rodríguez-García R., Garriga-Baraut T., Fernández-Rivas M., García-Figueroa B.E. (2015). Alérgenos alimentarios. Tratado de Alergología.

[10] Gelis S., Rueda M., Valero A., Fernández E.A., Moran M., Fernández-Caldas E. (2020). Shellfish allergy: Unmet needs in diagnosis and treatment. J. Investig. Allergol. Clin. Immunol..

[11] Santos A.F., Riggioni C., Agache I., Akdis C.A., Akdis M., Álvarez-Perea A., Alvaro-Lozano M., Ballmer-Weber B., Barni S., Beyer K. (2023). EAACI guidelines on the diagnosis of IgE-mediated food allergy. Allergy.

[12] Khora S.S. (2016). Seafood-Associated Shellfish Allergy: A Comprehensive Review. Immunol. Investig..

[13] Azofra J., Echechipia S., Irazábal B., Muñoz D., Bernedo N., Gacía B.E., Gastaminza G., Goikoetxea M.J., Joral A., Lasa E. (2017). Heterogenecity in allergy to mollusks: A clinical-immunological study in a population from the north of Spain. J. Investig. Allergol. Clin. Immunol..

[14] Steve L. (2008). Taylor. Molluscan Shellfish Allergy. Advances in Food and Nutrition Research.

[15] Wong L., Tham E.H., Lee B.W. (2019). An update on shellfish allergy. Curr. Opin. Allergy Clin. Immunol..

[16] Burney P., Summers C., Chinn S., Hooper R., Van Ree R., Lidholm J. (2010). Prevalence and distribution of sensitization to foods in the European Community Respiratory Health Survey: A EuroPrevall analysis. Allergy.

[17] Osterballe M., Hansen T.K., Mortz C.G., Høst A., Bindslev-Jensen C. (2005). The prevalence of food hypersensitivity in an unselected population of children and adults. Pediatr. Allergy Immunol..

[18] Alergológica 2015, SEAIC. https://www.seaic.org/inicio/noticias-general/alergologica-2015.html.

[19] Wai C.Y., Leung N.Y., Leung A.S., Wong G.W., Leung T.F. (2021). Seafood allergy in Asia: Geographical specificity and beyond. Front. Allergy.

[20] Ruethers T., Taki A.C., Johnston E.B., Nugraha R., Le T.T., Kalic T., Kalic T., McLean T.R., Kamath S.D., Lopata A.L. (2018). Seafood allergy: A comprehensive review of fish and shellfish allergens. Mol. Immunol..

[21] Tuano K.T.S., Davis C.M. (2018). Oral allergy syndrome in shrimp and house dust mite allergies. J. Allergy Clin. Immunol. Pract..

[22] Wang H.T., Warren C.M., Gupta R.S., Davis C.M. (2020). Prevalence and Characteristics of Shellfish Allergy in the Pediatric Population of the United States. J. Allergy Clin. Immunol. Pract..

[23] Sopo S.M., Monaco S., Badina L., Barni S., Longo G., Novembre E., Viola S., Monti G. (2015). Food protein-induced enterocolitis syndrome caused by fish and/or shellfish in Italy. Pediatr. Allergy Immunol..

[24] Ayuso R., Sánchez-Garcia S., Lin J., Fu Z., Ibáñez M.D., Carrillo T., Blanco C., Goldis M., Bardina L., Sastre J. (2010). Greater epitope recognition of shrimp allergens by children than by adults suggests that shrimp sensitization decreases with age. J. Allergy Clin. Immunol..

[25] La Bella G., Martella V., Basanisi M.G., Nobili G., Terio V., La Salandra G. (2017). Food-Borne viruses in shellfish: Investigation on novovirus and HAV presence in Apulia (SE Italy). Food Environ. Virol..

[26] Lopata A.L., Zinn C., Potter P.C. (1997). Characteristics of hypersensitivity reactions and identification of a unique 49 kd IgE-binding protein (Hal-m-1) in abalone (*Haliotis midae*). J. Allergy Clin. Immunol..

[27] De la Cuesta C.G., García B.E., Córdoba H., Diéguez I., Oehling A. (1989). Food allergy to Helix terrestre (snail). Allergol. Immunopathol..

[28] Van Ree R., Antonicelli L., Akkerdaas J.H., Pajno G.B., Barberio G., Corbetta L., Ferro G., Zambito M., Garritani M.S., Aalberse R.C. (1996). Asthma after consumption of snails in house-dust-mite allergic patients: A case of IgE cross-reactivity. Allergy.

[29] Martins L.M.L., Peltre G., da Costa Faro C.J.F., Vieira Pires E.M., da Cruz Inácio F.F. (2005). The Helix aspersa (brown garden snail) allergen repertoire. Int. Arch. Allergy Immunol..

[30] Carrillo T., De Castro F.R., Cuevas M., Caminero J., Cabrera P. (1991). Allergy to limpet. Allergy.

[31] Azofra J., Lombardero M. (2003). Limpet anaphylaxis: Cross-reactivity between limpet and house dust mite Dermatophagoides pteronyssinus. Allergy.

[32] Mederos-Luis E., Poza-Guedes P., Martínez M.J., González-Pérez R., Galán T., Sánchez-Machín I. (2023). Limpet molecular profile: Tropomyosin or not tropomyosin, that is the question. Thematic poster session (TPS). Allergy.

[33] Palma Carlos A.G., Migueis Clode M.E., Inacio F.F. Asthme Par Ingestion D’escargots. Allergie et Immunologie 1985 Tome 17, 5–6. https://atencionprimaria.almirallmed.es/wp-content/uploads/sites/12/2019/08/librito-alergias-alimentos.pdf.

[34] Guilloux L., Vuitton D.A., Delbourg M., Lagier A., Adessi B., Marchand C.R., Ville G. (1998). Cross-reactivity between terrestrial snails (*Helix species*) and house-dust mite (*Dermatophagoides pteronyssinus*). II. In vitro study. Allergy.

[35] Morikawa A., Kato M., Tokuyama K., Kuroume T., Minoshima M., Iwata S. (1990). Anaphylaxis to grand keyhole limpet (abalone-like shellfish) and abalone. Ann. Allergy.

[36] Carrillo T., Rodríguez de Castro F., Blanco C., Castillo R., Quiralte J., Cuevas M. (1994). Anaphylaxis due to limpet ingestión. Ann. Allergy.

[37] Gutierrez-Fernandez D., Fuentes-Vallejo M.S., Zavala B.B., Foncubierta-Fernandez A., Lucas-Velarde J., Leon-Jimenez A. (2009). Urticaria-angioedema due to limpet ingestion. J. Investig. Allergol. Clin. Immunol..

[38] Asero R., Scala E., Villalta D., Pravettoni V., Arena A., Billeri L., Colombo G., Cortellini G., Cucinelli F., De Cristofaro M.L. (2017). Shrimp Allergy: Analysis of Commercially Available Extracts for In Vivo Diagnosis. J. Investig. Allergol. Clin. Immunol..

[39] Heinzerling L., Mari A., Bergmann K.C., Bresciani M., Burbach G., Darsow U., Durham S., Fokkens W., Gjomarkaj M., Haahtela T. (2013). The skin prick test- European standards. Clin. Transl. Allergy.

[40] Gelis S., Rueda M., Pascal M., Fernández-Caldas E., Fernández E.A., Araujo-Sánchez G., Bartra J., Valero A. (2022). Usefulness of the nasal allergen provocation test in the diagnosis of shellfish allergy. J. Investig. Allergol. Clin. Immunol..

[41] Buyuktiryaki B., Masini M., Mori F., Barni S., Liccioli G., Sarti L., Lodi L., Giovannini M., du Toit G., Lopata A.L. (2021). IgE-Mediated Fish Allergy in Children. Medicina.

[42] Chong Neto H.J., Rosário N.A. (2009). Studying specific IgE: In vivo or in vitro. Allergol. Immunopathol..

[43] Hiller R., Laffer S., Harwanegg C., Huber M., Schmidt W.M., Twardosz A., Barletta B., Becker W.M., Blaser K., Breiteneder H. (2002). Microarrayed allergen molecules: Diagnostic gatekeepers for allergy treatment. FASEB J..

[44] Lupinek C., Wollmann E., Baar A., Banerjee S., Breiteneder H., Broecker B.M., Bublin M., Curin M., Flicker S., Garmatiuk T. (2014). Advances in allergen microarray technology for diagnosis and monitoring of allergy: The MeDALL allergen-chip. Methods.

[45] Keshavarz B., Platts-Mills T.A.E., Wilson J.M. (2021). The use of microarray and other multiplex technologies in the diagnosis of allergy. Ann. Allergy Asthma Immunol..

[46] Lis K., Bartuzi Z. (2023). Selected Technical Aspects of Molecular Allergy Diagnostics. Curr. Issues Mol. Biol..

[47] Leung P.S., Chen Y.C., Mykles D.L., Chow W.K., Li C.P., Chu K.H. (1998). Molecular identification of the lobster muscle protein tropomyosin as a seafood allergen. Mol. Mar. Biol. Biotechnol..

[48] Yu C., Gao X., Lin H., Xu L., Ahmed I., Khan M.U., Xu M., Chen Y., Li Z. (2020). Purification, Characterization, and Three-Dimensional Structure Prediction of Paramyosin, a Novel Allergen of Rapana venosa. J. Agric. Food Chem..

[49] Yun X., Li M.S., Chen Y., Huan F., Cao M.J., Lai D., Liu G.M. (2022). Characterization, Epitope Identification, and Cross-reactivity Analysis of Tropomyosin: An Important Allergen of Crassostrea angulata. J. Agric. Food Chem..

[50] Huan F., Han T.J., Liu M., Li M.S., Yang Y., Liu Q.M., Liu G.M. (2021). Identification and characterization of Crassostrea angulata arginine kinase, a novel allergen that causes cross-reactivity among shellfish. Food Funct..

[51] Han T.J., Liu M., Huan F., Li M.S., Xia F., Chen Y.Y., Liu G.M. (2020). Identification and Cross-reactivity Analysis of Sarcoplasmic-Calcium-Binding Protein: A Novel Allergen in Crassostrea angulata. J. Agric. Food Chem..

[52] Rolland J.M., Varese N.P., Abramovitch J.B., Anania J., Nugraha R., Kamath S., O’Hehir R.E. (2018). Effect of Heat Processing on IgE Reactivity and Cross-Reactivity of Tropomyosin and Other Allergens of Asia-Pacific Mollusc Species: Identification of Novel Sydney Rock Oyster Tropomyosin Sac g 1. Mol. Nutr. Food Res..

[53] Asturias J.A., Eraso E., Arilla M.C., Gómez-Bayón N., Inácio F., Martínez A. (2002). Cloning, isolation, and IgE-binding properties of *Helix aspersa* (brown garden snail) tropomyosin. Int. Arch. Allergy Immunol..

[54] Miyazawa H., Fukamachi H., Inagaki Y., Reese G., Daul C.B., Lehrer S.B., Inouye S., Sakaguchi M. (1996). Identification of the first major allergen of a squid (*Todarodes pacificus*). J. Allergy Clin. Immunol..

[55] Misnan R., Abd Aziz N.S., Yadzir Z.H.M., Bakhtiar F., Abdullah N., Murad S. (2016). Impacts of thermal treatments on major and minor allergens of sea snail (*Cerithidea obtuse*). Iran J. Allergy Asthma Immunol..

[56] Muddaluru V., Valenta R., Vrtala S., Schlederer T., Hindley J., Hickey P., Larché M., Tonti E. (2021). Comparison of house dust mite sensitization profiles in allergic adults from Canada, Europe, South Africa and USA. Allergy.

[57] Suzuki M., Kobayashi Y., Hiraki Y., Nakata H., Shiomi K. (2011). Paramyosin of the disc abalone Haliotis discus discus: Identification as a new allergen and cross-reactivity with tropomyosin. Food Chem..

[58] Scala E., Abeni D., Villella V., Villalta D., Cecchi L., Caprini E., Asero R. (2024). Investigating Novel Food Sensitization: A Real-Life Prevalence Study of Cricket, Locust, and Mealworm IgE-Reactivity in Naïve allergic Individuals. J. Investig. Allergol. Clin. Immunol..

[59] De Maat- Bleeker F., Akkerdaas J.H., Van Ree R., Aalberse R.C. (1995). Vineyard snail allergy possibly induced by sensitization to house-dust mite (*Dermatophagoides pteronyssinus*). Allergy.

[60] Prados-Castaño M., Cimbollek S., Bartolomé B., Castillo M., Quiralte J. (2024). Snail-induced anaphylaxis in patients with underlying Artemisia vulgaris pollinosis: The role of carbohydrates. Allergol. Immunopathol..

[61] Chung C.H., Mirakhur B., Chan E., Le Q.-T., Berlin J., Morse M., Murphy B.A., Satinover S.M., Hosen J., Mauro D. (2008). Cetuximab induced anaphylaxis and IgE specific for galactose-a-1,3-galactose. N. Engl. J. Med..

[62] Commins S.P., Satinover S.M., Hosen J., Mozena J., Borish L., Lewis B., Woodfolk J.A., Platts-Mills T.A. (2009). Delayed anaphylaxis, angioedema, or urticaria after consumption of red meat in patients with IgE antibodies specific for galactose-a-1,3-galactose. J. Allergy Clin. Immunol..

[63] Homann A., Schramm G., Jappe U. (2017). Glycans andglycan-specific IgE in clinical and molecular allergology: Sensitization, diagnostics and clinical symptoms. J. Allergy Clin. Immunol..

[64] Aalberse R.C., Akkerdaas J., Van Ree R. (2001). Cross-reactivity of IgE antibodies to allergens. Allergy.

